# Pregnancy outcomes in interferon-beta-exposed patients with multiple sclerosis: results from the European Interferon-beta Pregnancy Registry

**DOI:** 10.1007/s00415-020-09762-y

**Published:** 2020-02-26

**Authors:** Kerstin Hellwig, Yvonne Geissbuehler, Meritxell Sabidó, Catrinel Popescu, Alessandra Adamo, Joachim Klinger, Asher Ornoy, Peter Huppke, the European Interferon-beta Pregnancy Study Group, Metin Akbaba, Gustavo Borghesi, Joerg-Peter Bugge, Elke Detering, Evra Köfüncü, Claudia Luenzmann, Bettina Mueller, Axel Olivar, Kiliana Suzart-Woischnik, Eva-Maria Wicklein, Vanessa Beynon, Kate Brown, Nicholas Everage, Maria Naylor, Avni Pandhi, Anh Ly, Silke Scheller, Milorad Todorovic, Dominic Jack, Yvonne Samsinger, Richard Weitzman

**Affiliations:** 1 Department of Neurology, St. Joseph and St. Elisabeth Hospital, Ruhr University, Gudrunstraße 56, 44791 Bochum, Germany; 2 Global Medical Affairs, Novartis Pharma AG, Basel, Switzerland; 3 Research and Development, Global Epidemiology, Merck KGaA, Darmstadt, Germany; 4 Safety Surveillance and Aggregate Reports, Biogen Idec Ltd, Maidenhead, UK; 5 Global Safety, Bayer AG, São Paulo, Brazil; 6 Biometrics, Synteract GmbH, Munich, Germany; 7 Department of Medical Neurobiology, Hebrew University of Jerusalem, Jerusalem, Israel; 8 Department of Pediatrics and Pediatric Neurology, University Medical Center, Göttingen, Germany

**Keywords:** Multiple sclerosis, Interferon-beta, Disease-modifying drugs, Pregnancy, Congenital anomalies, Spontaneous abortions

## Abstract

**Background:**

Family planning is an important consideration for women with multiple sclerosis (MS), who are often diagnosed during their reproductive years. Currently, limited data are available on pregnancy outcomes in patients exposed to interferon-beta (IFN-beta) before or during pregnancy. Here, we present the cumulative pregnancy exposure data and prevalence of pregnancy and infant outcomes in IFN-beta-exposed pregnant women with MS from the European IFN-beta Pregnancy Registry.

**Methods:**

Using spontaneous and solicited reports, the registry collected data from 26 countries of the European Economic Area, consisting of information on women with MS identifying themselves to one of the Marketing Authorisation Holders (Bayer, Biogen, Merck KGaA, and Novartis) or healthcare professionals as pregnant and exposed to IFN-beta during pregnancy or within 1 month before conception. The outcomes collected by the registry included ectopic pregnancies, spontaneous abortions, elective terminations, live, and stillbirths with or without congenital anomalies. The prevalence of pregnancy outcomes was put in context with those reported in the general population.

**Results:**

Between 2009 and 2017, the registry collected 948 pregnancy reports with a known pregnancy outcome. Overall, 82.0% (777/948) of pregnancies resulted in live birth without congenital anomaly. When comparing IFN-beta-exposed pregnancies with the general population, the prevalence of spontaneous abortions (10.7% vs. 10–21%) and congenital anomalies in live births (2.1% vs. 2.1–4.1%) were found to be within reported ranges.

**Conclusions:**

The data gathered from these pregnancy cases suggest no evidence that IFN-beta exposure before conception and/or during pregnancy adversely increases the rate of congenital anomalies or spontaneous abortions.

**Supplementary Information:**

The online version of this article (10.1007/s00415-020-09762-y) contains supplementary material, which is available to authorized users.

## Introduction

Family planning is an important consideration for female patients with multiple sclerosis (MS) who are receiving treatment with disease modifying drugs (DMDs) that may be contraindicated in pregnancy. The introduction of DMDs for the treatment of MS over 20 years ago has improved clinical outcomes for many patients, yet studies at this time were limited and did not address concerns regarding the potential adverse effects of DMDs on pregnancy and infant outcomes [1]. Patients are, therefore, advised to discuss treatment options with their healthcare professional (HCP) prior to family planning, and in many instances, it may be recommended, or even required, for a woman to discontinue her treatment with DMDs during pregnancy planning or once pregnancy is confirmed.

There is currently no consensus in the literature regarding MS treatment up to and during pregnancy. Clinical treatment guidelines recommend that injectables such as interferon-beta (IFN-beta) are continued until pregnancy occurs; however, the current evidence to support this is considered weak based on the quality of evidence and risk–benefit balance [2–4]. There are some data, suggesting that exposure to IFN-beta during pregnancy may lead to an increased risk of lower mean birth weight, shorter mean birth length, and preterm birth (generally considered as a birth before 37 weeks of gestation) [5–8]. However, in recent years, several systematic reviews [5, 9, 10] and observational studies [6–8, 11–15] have shown no increase in adverse pregnancy outcomes in patients exposed to IFN-beta before or during pregnancy.

Data on the risks of IFN-beta use during pregnancy were limited at the time which the DMDs were launched by their respective pharmaceutical companies (Betaseron^®^, Bayer AG; Avonex^®^ and Plegridy^®^, Biogen; Rebif^®^, Merck KGaA; and Extavia^®^, Novartis Pharma AG). The prospective European IFN-beta Pregnancy Registry was initiated in 2009 at the request of the Committee for Medicinal Products for Human Use (CHMP), to address the lack of evidence on the safety of IFN-beta treatment before conception and during pregnancy. The registry was comprised of spontaneous and solicited reports from patient support programmes and pharmacovigilance databases conducted by the Market Authorisation Holders (MAHs), Bayer AG, Biogen, Merck KGaA, and Novartis Pharma AG.

Here, we present the final cumulative pregnancy exposure data and prevalence of pregnancy and infant outcomes in IFN-beta-exposed pregnant women from the European IFN-beta Pregnancy Registry.

## Patients and methods

### Study design

The European IFN-beta Pregnancy Registry was a prospective observational collection of data from women exposed to IFN-beta before and/or during pregnancy and their pregnancy outcomes. Data collected by each MAH from 26 countries within the European Economic Area (Supplementary Table 1) were transferred to a central repository for pooled analysis. Data on pregnancy exposure were acquired prior to the knowledge of pregnancy outcome, or prior to the detection of a congenital anomaly at prenatal examination. All patients included in the analysis were receiving either IFN-beta-1a (Avonex^®^, Plegridy^®^ or Rebif^®^) or IFN-beta-1b (Betaferon^®^ or Extavia^®^). All patients gave their informed consent prior to their inclusion in the analysis.

Data were collected from individual case-safety reports (ICSR) spontaneously reported to the pharmacovigilance databases and solicited reports from patient support programmes conducted by the four MAHs and reported to MAH pharmacovigilance databases. Prospective data from spontaneously reported cases on HCP-confirmed pregnancies with a confirmed diagnosis of MS were included in the registry between 01 April 2009 and 16 June 2017. In addition, after 2015, cases without a confirmed MS diagnosis, pregnancies not confirmed by an HCP, and solicited reports from prospectively identified patient support programmes were included in the registry.

### Study participants

Women who identified themselves to the MAHs or an HCP as being pregnant and who had been exposed to IFN-beta were eligible for this analysis. Data from women with a diagnosis of MS, who were pregnant and did not know the potential pregnancy outcome, and who had been prescribed one of the five approved IFN-beta therapies within 1 month before conception or at any time during pregnancy were included in the registry. Women were excluded from the registry if pregnancy outcome was known at the time of initial contact. Reports were followed prospectively for outcomes.

### Definition of exposure and outcomes

The start of pregnancy was defined as the first day of last menstrual period (LMP). Pregnant women prescribed IFN-beta were considered exposed if they received at least one prescription of IFN-beta at any dose within 1 month before conception or at any time during pregnancy. Timing of treatment exposure was classified by the latest trimester with exposure to IFN-beta; before conception (within 1 month before conception), first (0–13 weeks), second (14–26 weeks), third trimester (27–40 weeks), or timing unknown.

The possible pregnancy outcomes were ectopic pregnancy, spontaneous abortions, elective terminations, stillbirths, and live births with and without congenital anomalies.

Using the European Medicines Agency (EMA) guidelines [16], spontaneous abortions were defined as a pregnancy which spontaneously ended before 22 weeks of gestation, and included abortion, miscarriage, missed abortion, incomplete abortion, and early foetal death. Stillbirths were defined as a foetal death occurring after 22 weeks of pregnancy, further defined as foetal death in the uterus or during labour. A congenital anomaly was defined as a morphological functional and/or biomedical developmental disturbance in the embryo or foetus whether detected at birth or not.

### Statistical analysis

For the sample size calculation, it was estimated that at least 827 pregnancy cases exposed to IFN-beta with known pregnancy outcomes were required to detect a doubling in the prevalence of congenital anomalies compared with the general population (average reported prevalence rate = 3%) [17], with 80% power and 5% significance level.

Cases included in the registry were categorised into two groups according to their origin: (1) HCP-confirmed pregnancy reports received from an HCP, and (2) non-HCP-confirmed pregnancy reports received from a patient, either spontaneously or when solicited (i.e., provided by a patient support programme).

The prevalence of each study outcome, with the associated 95% confidence interval (CI), was estimated using the number of events divided by the total number of pregnancy cases with a known outcome. Elective terminations and stillbirths were classified according to the presence of foetal defects, and live births according to the presence of congenital anomalies following the EMA guidelines [16]. The prevalence of congenital anomalies was estimated among live births, still births, and elective terminations with known outcomes.

Pregnancy cases without outcome present were categorised by those with: (1) pending outcome, defined as no occurrence of birth at time of data lock, and (2) unknown outcome, defined as those who were lost to follow-up. Follow-up contact was made with the patients’ HCP to obtain outcome data 1 month after the expected delivery date. The time between expected delivery date and the first follow-up attempt was 1 month, similar to the interval between subsequent follow-up attempts. Cases were defined as ‘lost to follow-up’ when all follow-up attempts were made without success. For the ‘pending’ cases, no further attempt to contact the individual was made after three attempts, as described in the protocol, at which time they became lost to follow-up (hence ‘outcome unknown’).

The prevalence of pregnancy outcomes was calculated for the total number of cases, and as a sensitivity analysis for cases that met the original inclusion criteria, i.e., spontaneously reported cases of HCP-confirmed pregnancies where the mother also had a confirmed diagnosis of MS. These results were put in context with the general population using the published data [18–23].

Adverse events were reported in the infant/foetus cases (i.e., exposed to IFN-beta via the mother 1 month before and/or during pregnancy). Adverse events were coded using the Medical Dictionary of Regulatory Activities (MedDRA v21.1) preferred term in events occurring in all cases.

## Results

### Study participants and data

Between 01 April 2009 and 16 June 2017, a total of 2447 pregnancy reports were collected by the pharmacovigilance databases of the four MAHs, consisting of 1486 solicited reports and 961 spontaneous reports. These reports were further comprised of 312 spontaneous HCP-confirmed pregnancy cases with a confirmed diagnosis of MS, along with 2135 cases from patient support programmes with unconfirmed diagnosis of MS or an unconfirmed pregnancy.

**Fig. 1:**
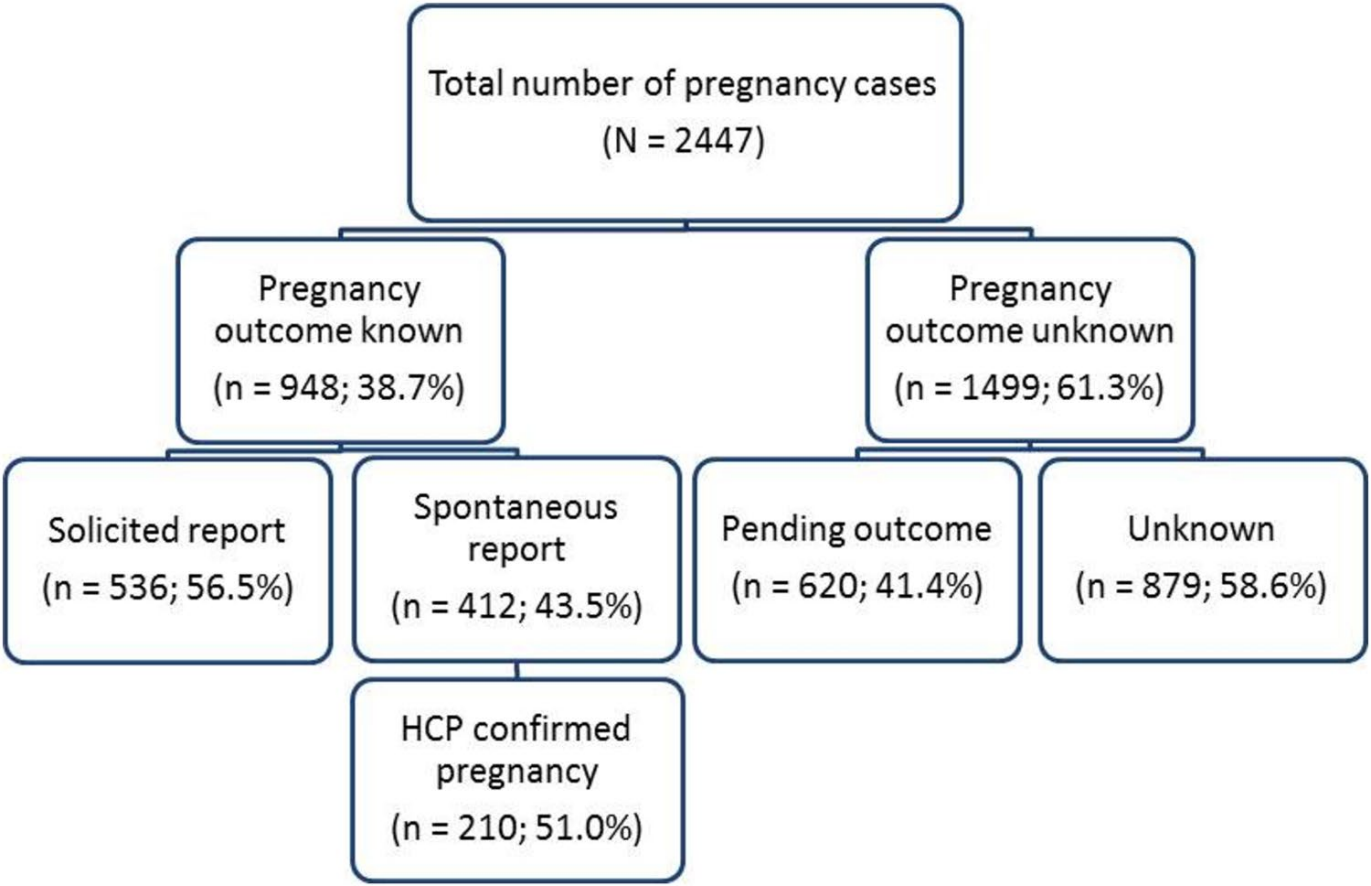
Total number of pregnancy cases by presence of outcome for all reported individual case safety reports

From the 2447 pregnancy reports collected, 948 (38.7%) cases had a known outcome, with the remaining cases either pending outcomes or patients that were lost to follow-up. Of the pregnancies with known outcome, 412 (43.5%) cases were spontaneously reported: with 210 (22.2%) cases of HCP-confirmed pregnancy, and 158 (16.7%) cases of HCP-confirmed pregnancy and a confirmed diagnosis of MS. Of the total number of pregnancies with known outcome, 795 (83.9%) cases had a confirmed diagnosis of MS (Fig. 1).

### Overall prevalence of pregnancy and infant outcomes

A total of 82.0% (777/948; 95% CI 79.36–84.36) of the pregnancy reports included in the European IFN-beta Pregnancy Registry had an outcome of live birth without congenital anomalies (Table 1). The remaining 18.0% of pregnancy reports are broken down as follows: ectopic pregnancies represented 0.4% (4/948; 95% CI 0.12–1.08); spontaneous abortions represented 10.7% (101/948; 95% CI 8.76–12.79); the prevalence of elective terminations was 0.6% (6/948; 95% CI 0.23–1.37) for those with foetal defects and 4.2% (40/948; 95% CI 3.03–5.70) without foetal defects, or unknown defects. Stillbirths with and without foetal defects had a prevalence of 0.1% (1/948; 95% CI 0.00–0.59) and 0.2% (2/948; 95% CI 0.03–0.76), respectively. Finally, the prevalence of live birth with any congenital anomalies was 1.8% (17/948; 95% CI 1.05–2.86).

**Table 1:** Cumulative number of individual case-safety reports and prevalence by pregnancy outcome and latest trimester of exposure to IFN-beta cases

Pregnancy or infant outcome, *n* (%)	Timing of IFN-beta exposure in pregnancy					Total (*n* = 948)	Exact 95% CI
	Before conception (*n* = 90)	First trimester (*n* = 573)	Second trimester (*n* = 23)	Third trimester (*n* = 8)	Timing unknown (*n* = 254)		
Ectopic pregnancies	0 (0.0)	1 (0.2)	0 (0.0)	0 (0.0)	3 (1.2)	4 (0.4)	0.12–1.08
Spontaneous abortion*	2 (2.2)	53 (9.2)	0 (0.0)	1 (12.5)	45 (17.7)	101 (10.7)	8.76–12.79
Elective termination (foetal defects)	2 (2.2)	4 (0.7)	0 (0.0)	0 (0.0)	0 (0.0)	6 (0.6)	0.23–1.37
Elective termination (no foetal defects/unknown)	2 (2.2)	23 (4.0)	0 (0.0)	0 (0.0)	15 (5.9)	40 (4.2)	3.03–5.70
Stillbirth with foetal defects	0 (0.0)	1 (0.2)	0 (0.0)	0 (0.0)	0 (0.0)	1 (0.1)	0.00–0.59
Stillbirth without foetal defects	0 (0.0)	1 (0.2)	1 (4.3)	0 (0.0)	0 (0.0)	2 (0.2)	0.03–0.76
Live birth with congenital anomaly	2 (2.2)	8 (1.4)	1 (4.3)	0 (0.0)	6 (2.4)	17 (1.8)	1.05–2.86
Live birth without congenital anomaly	82 (91.1)	482 (84.1)	21 (91.3)	7 (87.5)	185 (72.8)	777 (82.0)	79.36–84.36

*CI* confidence interval, *IFN-beta* interferon-beta

*Spontaneous abortion defined as when the pregnancy spontaneously ends before 22 completed weeks of gestation (< 24 weeks from last mentsrual period). This comprises spontaneous abortion, miscarriage, missed abortion, incomplete abortion, and early foetal death

**Fig. 2:**
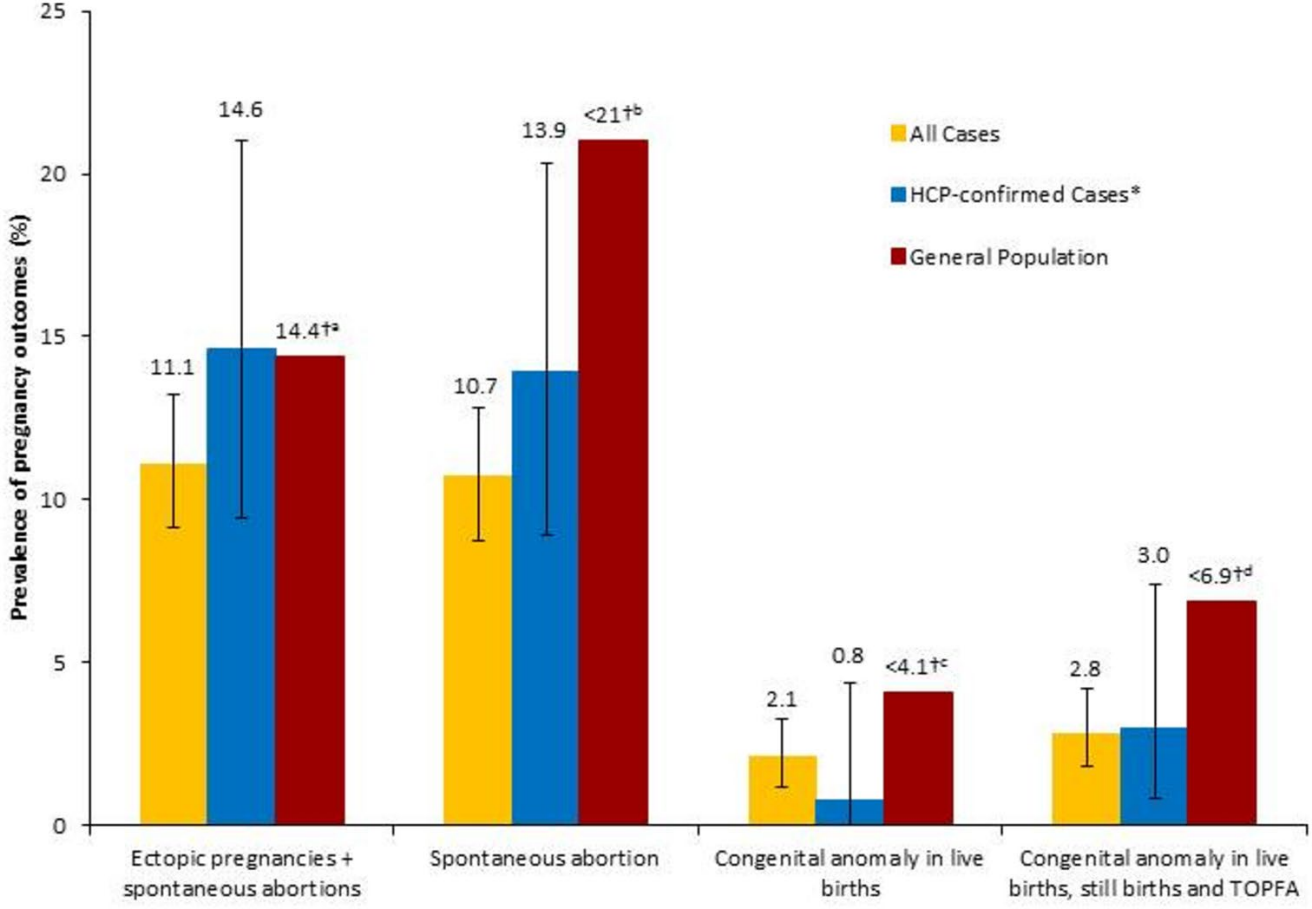
Prevalence of pregnancy and infant outcomes in women exposed to IFN-beta: all cases, in HCP-confirmed cases of pregnancy with confirmed diagnosis of MS, and the general population. Prevalence data are shown with 95% confidence interval for each population from the European IFN-beta Pregnancy Registry. *HCP-confirmed spontaneously reported pregnancy cases with confirmed diagnosis of MS. ^†^Values denote the maximum estimate from the general population as obtained from available literature. ^a^Wang et al. [23]; ^b^Buss et al. [18]; ^c^Congenital Malformations Registry [21]; ^d^Queisser-Luft et al. [22]. *HCP* healthcare professional, *MS* multiple sclerosis, *TOPFA* termination of pregnancy for foetal anomaly

The prevalence of spontaneous abortions and congenital anomalies in live births remained within the ranges reported in the general population (10.7% vs. 10–21% [18], and 2.1% vs. 2.1–4.1%, respectively) [19, 21]. This was also the case for the prevalence of congenital anomalies in live births, still births, and elective terminations (2.8% vs. 2.6–6.9%) [22] (Fig. 2).

### Prevalence of pregnancy and infant outcomes by timing of exposure

The latest timing of exposure was known in 73.2% (694/948) cases (Table 1). Among those cases, the majority of pregnancies were last exposed to IFN-beta before conception (13.0%; 90/694) or during the first trimester (82.6%; 573/694), with only a few pregnancies last exposed to IFN-beta during the second and third trimesters (3.3%; 23/694 and 1.2%; 8/694, respectively). The mean duration of exposure during pregnancy was 4.31 weeks with a standard deviation of 5.44 (Supplementary Table 1).

The prevalence of live births with congenital anomalies among the 573 cases with known outcomes and with exposure to IFN-beta during the first trimester was 1.4% (8/573). Exposure during the second or third trimesters occurred in 31 cases with a known outcome; of these cases, there were three reports of abnormal pregnancy outcomes: one stillbirth without foetal defects, one live birth with congenital anomaly (both cases were exposed during the second trimester), and one spontaneous abortion (reported during the third trimester, despite not being in accordance with the EMA definition of spontaneous abortion).

### Sensitivity analysis of pregnancy and infant outcomes by source of case

A sensitivity analysis was conducted to evaluate the differences between the spontaneous and solicited case reports. This analysis found that in the spontaneously reported population, the prevalence of live births with congenital anomaly was 0.7% (3/412; 95% CI 0.15–2.11), still birth with foetal defects was 0.5% (2/412; 95% CI 0.06–1.74), and elective terminations with foetal defects was 1.0% (4/412; 95% CI 0.27–2.47) (Supplementary Table 2a). In the solicited population, the prevalence of live births with congenital anomaly was 2.6% (14/536; 95% CI 1.44–4.34); still birth with foetal defects was 0.2% (1/412; 95% CI 0.00–0.69); and elective terminations with foetal defects were 0.4% (2/412; 95% CI 0.05–1.34) (Supplementary Table 2b), thus, indicating there are a few differences in the distribution of outcomes between the spontaneous versus solicited populations.

### Adverse events in infants exposed to IFN-beta before and/or during pregnancy

A total of 216 adverse events, including low birth weight and premature births, were reported in 98 infant/foetus cases exposed to IFN-beta via the mother during pregnancy. A broad range of adverse events were collected in the registry; however, no patterns of congenital anomalies were observed.

The congenital anomalies in cases of live birth (term births, preterm births, and not specified), still birth, and elective termination are described in Table 2.

**Table 2:** Congenital anomalies in cases of live birth (term births, preterm births, and not specified), still birth and elective termination

Case number	Pregnancy outcome	Congenital anomalies/foetal disorder
1	Term live birth	Persistent foetal circulation
2	Term live birth	Congenital ureterovesical junction anomaly Congenital urinary tract obstruction Congenital vesicoureteric reflux Ectopic kidney Renal aplasia
3	Term live birth	Developmental hip dysplasia
4	Term live birth	Developmental hip dysplasia
5	Term live birth	Cryptorchism
6	Term live birth	Ventricular septal defect
7	Term live birth	Congenital joint malformation
8	Term live birth	Ventricular septal defect
9	Term live birth	Foetal distress syndrome
		Hypoxic-ischaemic encephalopathy
		Foetal growth restriction
		Cerebral ischaemia
10	Not specified live birth	Developmental hip dysplasia
11	Not specified live birth	Cleft palate
12	Not specified live birth	Cerebral cyst
		Heart disease congenital
		Thymus hypoplasia
13	Not specified live birth	Ventricular septal defect
14	Not specified live birth	Heart disease congenital
15	Preterm live birth	Vocal cord paresis
16	Preterm live birth	Trisomy 21
		Duodenal atresia
17	Preterm live birth	Asphyxia
		Renal failure neonatal
18	Elective termination	Trisomy 21
19	Elective termination	Anencephaly
20	Elective termination	Eagle Barrett syndrome
21	Elective termination	Congenital renal disorder
22	Elective termination	Foetal disorder
23	Elective termination	Foetal growth restriction
24	Stillbirth	Foetal defects

## Discussion

Between 2009 and 2017, a total of 948 pregnancy cases with known outcomes were identified in which the prevalence of spontaneous abortions, congenital anomalies in live births and live births, still births, and elective terminations remained within the ranges reported in the general population (10.7% vs. up to 10–21% [18], 2.1% vs. 2.1–4.1% [19, 21] and 2.8% vs. 2.6–6.9% [22], respectively) (Fig. 2). This similarity was also observed when considering only the HCP-confirmed, spontaneously reported cases with a confirmed diagnosis of MS. A number of systematic literature reviews and observational studies have been published on the effect of IFN-beta exposure during pregnancy and the related pregnancy outcomes. Overall, the rates of congenital anomalies and spontaneous abortions reported in these studies are comparable with the rates reported in the general population [5, 9]. The estimated prevalence of pregnancy outcomes among IFN-beta-exposed patients, collected by the European IFN-beta Pregnancy Registry, in all cases including those that were spontaneously reported cases of HCP-confirmed pregnancies with a confirmed diagnosis of MS, remained within the ranges reported for the general population. Based on these results, there is no evidence of a signal, suggesting that exposure to IFN-beta before conception and/or during the first trimester of pregnancy adversely increases the rate of congenital anomalies or spontaneous abortions. Sensitivity analyses showed that the pregnancy outcomes of the cases included after 2015 were comparable between spontaneous and solicited cases (Fig. 2).

The results presented in Table 1 show that in the majority of pregnancy cases, IFN-beta treatment was stopped during the first trimester, a time at which most women become aware that they are pregnant, and that there is no evidence of a signal suggesting exposure to IFN-beta during this period adversely affects pregnancy or infant outcomes. Similar results have been shown in other observational studies, where exposure in the early stages of pregnancy has not been shown to worsen pregnancy outcomes [7, 8, 12, 14]. The European IFN-beta Pregnancy Registry collected a limited number of pregnancies exposed to IFN-beta during the second and third trimesters (3.3%; 31/948), so it is not possible to determine the impact of IFN-beta exposure during the latter stages of pregnancy; however, exposure occurred at a critical time in organ development [24]. In previous studies, the timing of exposure to IFN-beta was also relatively narrow, with a very low number of pregnancies being exposed to IFN-beta beyond the first trimester. Mean duration of foetal exposure time to IFN-beta ranged from 4.0 to 9.1 weeks [5–8, 12, 14]. It should be noted that Table 1 presents one spontaneous abortion that occurred during the third trimester. This may be due to an error in which a pregnancy case was incorrectly reported to the pharmacovigilance database, and was not possible to be clarified during follow-up. This particular case is not in accordance with the EMA definition of spontaneous abortion and should, therefore, be disregarded.

The European IFN-beta Pregnancy Registry reported a total of 216 adverse events in 98 infant/foetus cases, yet no patterns of congenital anomalies were observed. These findings align with previous studies which found that in cases in which congenital anomalies were present, they did not centre on a specific system organ class [6, 11, 13, 14]. The cases of adverse events were reported retrospectively to the pharmacovigilance databases, and as such, 11 cases could not be matched to the mother’s case report. It should also be noted that some adverse events in infant/foetus cases may not have been reported to the pharmacovigilance databases due to the case being lost to follow-up.

The number of pregnancy reports from patients who met the initial inclusion criteria did not reach the estimated number of cases required to detect a doubling in outcome prevalence. Therefore, after June 2015, patients with an unconfirmed diagnosis of MS, solicited reports from patient support programmes and pregnancy cases not confirmed by an HCP were permitted to be included in the analysis. Through the inclusion of these cases, the required number of pregnancy reports, calculated to be 827, was exceeded by 121 pregnancy cases. Sensitivity analyses indicated that the inclusion of the additional non-HCP-confirmed and solicited cases were comparable to the pregnancy outcomes of the cases meeting the original inclusion criteria, and were, therefore, deemed adequate as a measure to reach the required sample size for the analysis. A limitation of this analysis is that 16.1% of patients with known pregnancy outcome did not have a confirmed diagnosis of MS; however, in Europe, treatment with IFN-beta automatically means that the diagnosis of MS is confirmed by an HCP.

At the end of the study period, approximately 61.3% of the total numbers of reported cases were either pending outcomes (25.3%) or the patients were lost to follow-up (35.9%); as such, there may be selection bias for pregnancies with documented outcomes versus those that were not documented. This selection bias may be either towards the presence of adverse pregnancy outcomes or towards a particular subgroup of women who have higher healthcare seeking behaviour. It is also thought that the seriousness of an adverse event contributes to whether or not it is reported, with serious events more likely to be reported than non-serious events [25]. However, it may also be possible that the mothers participating in the pregnancy registry may be healthier, and therefore, the rate of adverse events would be expected to be lower.

The European IFN-beta Pregnancy Registry did not separate the pregnancy outcomes by treatment type (i.e., IFN-beta-1a and beta-1b) to determine the adverse pregnancy outcomes associated with each treatment. However, if the results were separated for analysis, then the statistical power within each treatment group would be limited and meaningful conclusions could not be drawn from the data. Due to the design of the study, it was not possible to determine the dose or duration of IFN-beta exposure prior to conception and pregnancy. Therefore, it cannot be determined if pregnancies with congenital anomalies were associated with higher doses of IFN-beta, longer term DMD use, a combination of these factors, or whether adverse pregnancy outcomes occur regardless of the dosage and length of exposure. It should also be noted that the European IFN-Beta Pregnancy Registry did not adjust for potential confounders including family history, gestational age, parity, previous obstetrical history, smoking and alcohol consumption, comorbidities, or exposure to co-medications, which may have influenced the prevalence results. Nor did the study collect any substantial data on birth weight, birth length, or gestational age at birth to allow for a comprehensive analysis. Furthermore, 375 (54%) of the 694 cases reported in this study were obtained from patients in Germany; therefore, interpretations should take into consideration the differences in clinical practices between Germany and the rest of the countries participating in this study. Nevertheless, the available data do not suggest any concern regarding the ability to extrapolate the results to the intended target population.

The European IFN-beta Pregnancy Registry was initiated at the request of the CHMP, and was justified by the need to provide HCPs and women of childbearing age with MS additional information on IFN-beta exposure during pregnancy, so that informed treatment decisions can be made. Overall, the results from the European IFN-beta Pregnancy Registry represent the largest prospective cohort providing safety data of women with MS of childbearing age exposed to IFN-beta. Results from the registry showed no evidence that IFN-beta exposure before conception and/or during pregnancy increased the rate of congenital anomalies or spontaneous abortions, thus indicating that treatment with IFN-beta may be continued until pregnancy is confirmed.

## Electronic supplementary material

Below is the link to the electronic supplementary material.

Supplementary file1 (DOCX 20 kb)

Supplementary file2 (DOCX 24 kb)
