# Supplementary material for: Pregnancy outcomes in interferon-beta-exposed patients with multiple sclerosis: results from the European Interferon-beta Pregnancy Registry
Source: J Neurol. 2020 Feb 26;267(6):1715–23. doi: 10.1007/s00415-020-09762-y (PMC7293672; doi:10.1007/s00415-020-09762-y)
Supplement: Supplementary file 1 — Supplementary file1 (DOCX 20 kb) [file 415_2020_9762_MOESM1_ESM.docx]

Supplementary Table 1. Source of information and exposure during pregnancy by country for cases with known pregnancy outcome

|  | Solicited  (n = 362) | Spontaneous  (n = 332) | Total  (N = 694) | Cumulative duration of exposure during pregnancy [weeks] over all cases | Average duration of exposure during pregnancy [weeks] per case | % of cases with exact timing of exposure unknown |
| --- | --- | --- | --- | --- | --- | --- |
| Country | | | | | | |
| Total (%) | 362 (100.0) | 332 (100.0) | 694 (100.0) | 1602.6 | 4.31 (SD = 5.44) | 46.4 |
| Austria | 7 ( 1.9) | 0 ( 0.0) | 7 ( 1.0) | 71.6 | 11.93 | 14.3 |
| Belgium | 26 ( 7.2) | 7 ( 2.1) | 33 ( 4.8) | 79.0 | 8.78 | 72.7 |
| Bulgaria | 2 ( 0.6) | 0 ( 0.0) | 2 ( 0.3) | 3.7 | 3.71 | 50.0 |
| Cyprus | 0 ( 0.0) | 3 ( 0.9) | 3 ( 0.4) | 7.4 | 2.48 | 0.0 |
| Czech Republic | 14 ( 3.9) | 9 ( 2.7) | 23 ( 3.3) | 33.1 | 2.37 | 39.1 |
| Denmark | 0 ( 0.0) | 2 ( 0.6) | 2 ( 0.3) | 19.3 | 19.29 | 50.0 |
| Estonia | 1 ( 0.3) | 0 ( 0.0) | 1 ( 0.1) | . | . | 100.0 |
| Finland | 1 ( 0.3) | 0 ( 0.0) | 1 ( 0.1) | . | . | 100.0 |
| France | 10 ( 2.8) | 15 ( 4.5) | 25 ( 3.6) | 71.1 | 4.74 | 40.0 |
| Germany | 187 ( 51.7) | 188 ( 56.6) | 375 ( 54.0) | 847.1 | 4.11 | 45.1 |
| Greece | 18 ( 5.0) | 14 ( 4.2) | 32 ( 4.6) | 87.1 | 3.63 | 25.0 |
| Hungary | 1 ( 0.3) | 9 ( 2.7) | 10 ( 1.4) | 21.4 | 3.57 | 40.0 |
| Iceland | 0 ( 0.0) | 1 ( 0.3) | 1 ( 0.1) | 3.9 | 3.86 | 0.0 |
| Ireland | 8 ( 2.2) | 7 ( 2.1) | 15 ( 2.2) | 30.4 | 3.80 | 46.7 |
| Italy | 13 ( 3.6) | 4 ( 1.2) | 17 ( 2.4) | 41.0 | 2.93 | 17.6 |
| Latvia | 3 ( 0.8) | 0 ( 0.0) | 3 ( 0.4) | 23.3 | 11.64 | 33.3 |
| Luxembourg | 0 ( 0.0) | 1 ( 0.3) | 1 ( 0.1) | . | . | 100.0 |
| Netherlands | 18 ( 5.0) | 11 ( 3.3) | 29 ( 4.2) | 55.4 | 4.26 | 55.2 |
| Poland | 12 ( 3.3) | 10 ( 3.0) | 22 ( 3.2) | 49.6 | 4.13 | 45.5 |
| Portugal | 23 ( 6.4) | 4 ( 1.2) | 27 ( 3.9) | 61.4 | 4.10 | 44.4 |
| Romania | 1 ( 0.3) | 0 ( 0.0) | 1 ( 0.1) | 3.1 | 3.14 | 0.0 |
| Slovakia | 1 ( 0.3) | 6 ( 1.8) | 7 ( 1.0) | 6.4 | 2.14 | 57.1 |
| Slovenia | 3 ( 0.8) | 0 ( 0.0) | 3 ( 0.4) | . | . | 100.0 |
| Spain | 8 ( 2.2) | 5 ( 1.5) | 13 ( 1.9) | 9.7 | 2.43 | 69.2 |
| Sweden | 2 ( 0.6) | 4 ( 1.2) | 6 ( 0.9) | 8.7 | 2.90 | 50.0 |
| United Kingdom | 3 ( 0.8) | 32 ( 9.6) | 35 ( 5.0) | 68.6 | 6.23 | 68.6 |
