# Supplementary material for: Pregnancy outcomes in interferon-beta-exposed patients with multiple sclerosis: results from the European Interferon-beta Pregnancy Registry
Source: J Neurol. 2020 Feb 26;267(6):1715–23. doi: 10.1007/s00415-020-09762-y (PMC7293672; doi:10.1007/s00415-020-09762-y)
Supplement: Supplementary file 2 — Supplementary file2 (DOCX 24 kb) [file 415_2020_9762_MOESM2_ESM.docx]

Table 2a: Cumulative number of individual case safety reports and prevalence by pregnancy outcome and latest trimester of exposure to IFN-beta - spontaneous reports only

| **Pregnancy or infant outcome** | **Timing of IFN-beta exposure in pregnancy** | | | | | | **Exact  95% CI** |
| --- | --- | --- | --- | --- | --- | --- | --- |
|  | **Before conception (n = 46)** | **First trimester (n = 270)** | **Second trimester (n = 13)** | **Third trimester (n = 3)** | **Timing unknown  (n = 80)** | **Total  (N = 412)** |  |
| Ectopic pregnancies, n (%) | 0 (0.0) | 0 (0.0) | 0 (0.0) | 0 (0.0) | 1 (1.3) | 1 (0.2) | 0.01–1.34 |
| Spontaneous abortion, n (%) | 1 (2.2) | 24 (8.9) | 0 (0.0) | 0 (0.0) | 13 (16.3) | 38 (9.2) | 6.61–12.44 |
| Elective termination (foetal defects), n (%) | 2 (4.3) | 2 (0.7) | 0 (0.0) | 0 (0.0) | 0 (0.0) | 4 (1.0) | 0.27–2.47 |
| Elective termination (no foetal defects/unknown), n (%) | 1 (2.2) | 13 (4.8) | 0 (0.0) | 0 (0.0) | 9 (11.3) | 23 (5.6) | 3.57–8.26 |
| Stillbirth with foetal defects, n (%) | 0 (0.0) | 0 (0.0) | 0 (0.0) | 0 (0.0) | 0 (0.0) | 1 (0.1) | 0.00–0.89 |
| Stillbirth without foetal defects, n (%) | 0 (0.0) | 1 (0.4) | 1 (7.7) | 0 (0.0) | 0 (0.0) | 2 (0.5) | 0.06–1.74 |
| Live birth with congenital anomaly, n (%) | 1 (2.2) | 0 (0.0) | 0 (0.0) | 0 (0.0) | 2 (2.5) | 3 (0.7) | 0.15–2.11 |
| Live birth without congenital anomaly, n (%) | 41 (89.1) | 230 (85.2) | 12 (92.3) | 3 (100.0) | 55 (68.8) | 341 (82.8) | 78.77–86.29 |
|  | | | | | | | |

Table 2b: Cumulative number of individual case safety reports and prevalence by pregnancy outcome and latest trimester of exposure to IFN-beta - solicited reports only

| **Pregnancy or infant outcome** | **Timing of IFN-beta exposure in pregnancy** | | | | | | **Exact  95% CI** |
| --- | --- | --- | --- | --- | --- | --- | --- |
|  | **Before conception (n = 44)** | **First trimester (n = 303)** | **Second trimester (n = 10)** | **Third trimester (n = 5)** | **Timing unknown  (n = 174)** | **Total  (N = 536)** |  |
| Ectopic pregnancies, n (%) | 0 (0.0) | 1 (0.3) | 0 (0.0) | 0 (0.0) | 2 (1.1) | 3 (0.6) | 0.12–1.63 |
| Spontaneous abortion, n (%) | 1 (2.3) | 29 (9.6) | 0 (0.0) | 1 (20.0) | 32 (18.4) | 63 (1.8) | 9.15–14.79 |
| Elective termination (foetal defects), n (%) | 0 (0.0) | 2 (0.7) | 0 (0.0) | 0 (0.0) | 0 (0.0) | 2 (0.4) | 0.05–1.34 |
| Elective termination (no foetal defects/unknown), n (%) | 1 (2.3) | 10 (3.3) | 0 (0.0) | 0 (0.0) | 6 (3.4) | 17 (3.2) | 1.86–5.03 |
| Stillbirth with foetal defects, n (%) | 0 (0.0) | 1 (0.3) | 0 (0.0) | 0 (0.0) | 0 (0.0) | 1 (0.2) | 0.00–1.04 |
| Stillbirth without foetal defects, n (%) | 0 (0.0) | 0 (0.0) | 0 (0.0) | 0 (0.0) | 0 (0.0) | 0 (0.0) | 0.00–0.69 |
| Live birth with congenital anomaly, n (%) | 1 (2.3) | 8 (2.6) | 1 (10.0) | 0 (0.0) | 4 (2.3) | 14 (2.6) | 1.44–4.34 |
| Live birth without congenital anomaly, n (%) | 41 (93.2) | 252 (83.2) | 9 (90.0) | 4 (80.0) | 130 (74.7) | 436 (81.3) | 77.78–84.5 |
|  | | | | | | | |
